# GM1 ganglioside exerts protective effects against glutamate‐excitotoxicity via its oligosaccharide in wild‐type and amyotrophic lateral sclerosis motor neurons

**DOI:** 10.1002/2211-5463.13727

**Published:** 2023-11-15

**Authors:** Giulia Lunghi, Erika Di Biase, Emma Veronica Carsana, Alexandre Henriques, Noelle Callizot, Laura Mauri, Maria Grazia Ciampa, Luigi Mari, Nicoletta Loberto, Massimo Aureli, Sandro Sonnino, Michael Spedding, Elena Chiricozzi, Maria Fazzari

**Affiliations:** ^1^ Department of Medical Biotechnology and Translational Medicine University of Milano Segrate Italy; ^2^ Neuro‐sys Gardanne France; ^3^ Department of Immunology St. Jude Children's Research Hospital Memphis TN USA; ^4^ Spedding Research Solutions Le Vésinet France

**Keywords:** amyotrophic lateral sclerosis, excitotoxicity, GM1 ganglioside, GM1 oligosaccharide, intracellular aggregates, mitochondria

## Abstract

Alterations in glycosphingolipid metabolism have been linked to the pathophysiological mechanisms of amyotrophic lateral sclerosis (ALS), a neurodegenerative disease affecting motor neurons. Accordingly, administration of GM1, a sialic acid‐containing glycosphingolipid, is protective against neuronal damage and supports neuronal homeostasis, with these effects mediated by its bioactive component, the oligosaccharide head (GM1‐OS). Here, we add new evidence to the therapeutic efficacy of GM1 in ALS: Its administration to WT and *SOD1* ^*G93A*^ motor neurons affected by glutamate‐induced excitotoxicity significantly increased neuronal survival and preserved neurite networks, counteracting intracellular protein accumulation and mitochondria impairment. Importantly, the GM1‐OS faithfully replicates GM1 activity, emphasizing that even in ALS the protective function of GM1 strictly depends on its pentasaccharide.

## Graphical abstract

We provide evidence for the ability of GM1‐oligosaccharide (GM1‐OS) to prevent the neurodegeneration of an *in vitro* model of amyotrophic lateral sclerosis (ALS) by acting on crucial processes of disease pathophysiology (excitotoxicity, SOD1 aggregation, TDP43 mislocalization, mitochondria dysfunction, and oxidative stress). This evidence opens a novel therapeutic perspective for ALS treatment.

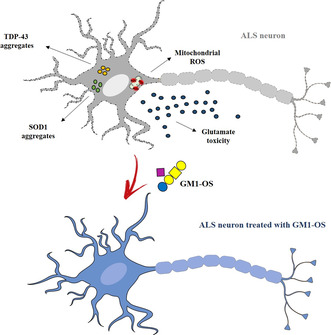

**Abbreviations**
Ganglioside nomenclature: in accordance with IUPAC‐IUBB recommendations
ALS: amyotrophic lateral sclerosis
AMPA: amino‐3‐hydroxy‐5‐methyl‐4‐isoxazolepropionic acid
BDNF: brain derived neurotrophic factor
BSA: bovine serum albumin
*C9orf72*
: chromosome 9 open reading frame 72
CNS: central nervous system
CTRL: control
DCB: dichlorobenzamil
DMEM: Dulbecco's modified Eagles' medium
ERK1/2: extracellular signal‐regulated protein kinases 1 and 2
FBS: fetal bovine serum
FCS: fetal calf serum
Glut: glutamate
GM1: II^3^Neu5Ac‐Gg_4_Cer, β‐Gal‐(1–3)‐β‐GalNAc‐(1–4)‐[α‐Neu5Ac‐(2–3)]‐β‐Gal‐(1–4)‐β‐Glc‐Cer
GM1‐OS: GM1 oligosaccharide, II^3^Neu5Ac‐Gg_4_, β‐Gal‐(1–3)‐β‐GalNAc‐(1–4)‐[α‐Neu5Ac‐(2–3)]‐β‐Gal‐(1–4)‐β‐Glc
HPTLC: high‐performance thin‐layer chromatography
MAP2: microtubule‐associated protein 2
MAPK: mitogen‐activated protein kinase
MNs: motor neurons
MPTP: 1‐methyl‐4‐phenyl‐1,2,3,6‐tetrahydropyridine hydrochloride
N2a: neuroblastoma Neuro2a cells
NGF: nerve growth factor
NMDA: *N*‐methyl‐d‐aspartate
$O_{2}^{\cdot -}$
: superoxide anion
PBS: phosphate‐buffered saline
ROS: reactive oxygen species
RRID: research resource identifiers
SOD1: superoxide dismutase 1
*SOD1* ^*G93A*^
: superoxide dismutase 1 with substitution of glycine (G) 93 to alanine (A)
TDP‐43: TAR DNA binding protein
TrkA: tropomyosin receptor kinase A
WT: wild‐type

In the central nervous system (CNS), glutamate is the main fast excitatory neurotransmitter orchestrating many fundamental brain processes, including synaptic plasticity events associated with memory and learning, neuronal networks formation, and CNS repair [1].

Glutamate release in the synaptic cleft stimulates ionotropic glutamate receptors (amino‐3‐hydroxy‐5‐methyl‐4‐isoxazolepropionic acid, AMPA and *N*‐methyl‐d‐aspartate, NMDA) on the postsynaptic neuron, leading to sodium and calcium influx, to depolarization, and finally to the generation of action potential [2]. However, excessive stimulation of the glutamate receptors triggers excitotoxicity, a process known to lead to neurodegeneration [2, 3]. Accordingly, an excess of calcium influx can determine an overload of mitochondria that depolarizes and increases reactive oxygen species (ROS) production [2, 3].

Among neuronal cells, motor neurons (MNs) are particularly susceptible to AMPA receptor‐mediated excitotoxicity, and this seems to be linked to their limited capability to buffer the calcium increase, due to low expression of calcium‐buffering proteins [2].

Accordingly, glutamate excitotoxicity is one of the main features of amyotrophic lateral sclerosis (ALS), a neurodegenerative disorder characterized by degeneration of both upper and lower MNs [4]. Around 5–10% of ALS forms are familial and four genes account for about two‐thirds of cases: chromosome 9 open reading frame 72 (*C9orf72*), superoxide dismutase 1 (*SOD1*), TAR DNA binding protein (*TARDBP*, encoding for TAR DNA‐binding protein‐43, TDP‐43), and fused in sarcoma (*FUS*) [5].

Among them, the most widely studied is *SOD1*, which encodes for copper/zinc ion‐binding superoxide dismutase and whose mutations determine a toxic gain of function [5, 6]. Interestingly, several pieces of evidence raise the possibility that *SOD1* mutations may increase the susceptibility of MNs to excitotoxicity [2]. Additionally, accumulating data indicate that mutant SOD1 protein localizes in mitochondria affecting their function as evidenced by the reduced activity of mitochondrial electron transport chain complexes and decreased ATP levels in the mutant *SOD1* ^*G93A*^ mouse model [7, 8]. Moreover, it has been demonstrated that mitochondrial localization of mutant SOD1 causes cytochrome c release activating the caspase cascade [9].

Growing evidence points to the possibility that abnormal changes in gangliosides homeostasis may play a role in the etiology of the ALS disease [10, 11, 12].

Gangliosides are sialic acid‐containing glycosphingolipids with important roles both in the structural organization of cell membranes and in the regulation of cell signaling [13, 14, 15, 16, 17]. Among them, the GM1 ganglioside is highly enriched in neuronal plasma membranes, and it has been described to regulate numerous neuronal activities aimed at maintaining neuronal homeostasis [18, 19, 20, 21, 22].

A series of studies proved GM1 protection of neuronal cultures exposed to excessive amounts of glutamate [23, 24, 25] and, notably, alterations in ganglioside content due to the lack of GM2/GD2 synthase increased the susceptibility to excitotoxicity‐induced neuronal death that was efficiently counteracted by the administration of GM1 or its synthetic‐derivative (LIGA20) both *in vitro* and *in vivo* [26, 27].

Accordingly, the levels of the spinal cord gangliosides and of the enzymes that regulate their metabolism are altered in ALS patients and animal models. Interestingly, Dodge *et al*. [10] demonstrated that the inhibition of gangliosides synthesis in *SOD1* ^*G93A*^ mice worsened the disease progression while the administration of GM3, the precursor of a‐series gangliosides, slowed it down.

Among the examined gangliosides, during the early symptomatic and end‐stage phases of ALS disease, GM1 levels were found to be significantly lower in the spinal cord of *SOD1* ^*G93A*^ mice as compared to wild‐type (WT) littermates [10]. Interestingly, exogenous administration of GM1 was able to reduce MNs death in adult rats after ventral roots avulsion [28], and to ameliorate pathological symptoms in patients suffering from spinal cord injury [18, 29].

As a membrane lipid, GM1 ganglioside is composed by a ceramide tail inserted in the outer layer of the cell membrane and an oligosaccharide chain (GM1‐OS) protruding in the extracellular space [18, 20, 21]. Our group has recently discovered that the oligosaccharide GM1‐OS is the GM1 bioactive portion, that, even when separated from the parental glycolipid, replicates all the protective and restorative functions of GM1, by interacting with the nerve growth factor (NGF) specific tropomyosin receptor kinase A (TrkA) at the cell surface [30]. As reported for GM1, the GM1‐OS/TrkA interaction activates a series of events including cell migration, clustering and adhesion, modulation of mitochondrial activity, and regulation of intracellular calcium fluxes [30].

In this work, we studied the potential protective effect of GM1 against glutamate induced‐excitotoxicity in both WT and *SOD1* ^*G93A*^ MNs, to understand whether this function is mediated by the GM1‐OS.

Our results indicate that GM1, via its oligosaccharide portion, increases neuronal survival reducing aggregated SOD1 levels and the mislocalization of TDP‐43, typical markers of ALS pathology, possibly by modulating the mitochondria machinery.

## Materials and methods

### Materials

Commercial chemicals were of the highest purity available, common solvents were distilled before use and water was doubly distilled in a glass apparatus. Cell culture plates were from Corning (Corning, NY, USA). Mouse neuroblastoma Neuro2a (N2a) cells (RRID: CVCL_0470), phosphate‐buffered saline (PBS), paraformaldehyde, Dako fluorescent mounting medium, bovine serum albumin (BSA), trypan blue, ethylenediaminetetraacetic acid, dimethyl sulfoxide, d‐glucose, Dulbecco's modified Eagle's (DMEM) high glucose medium, SYBR Green Extract‐N‐Amp tissue PCR kit, DNAse I grade II, glutamate, dichlorobenzamil (DCB), ethanol, acetic acid, saponin, Hoechst, sodium acetate, calcium chloride (CaCl_2_), *Vibrio cholerae* sialidase, methanol, trimethylamine, chloroform, 2‐propanol were from Sigma‐Aldrich (St. Louis, MO, USA). Fetal bovine serum (FBS), fetal calf serum (FCS), l‐glutamine, sodium pyruvate, penicillin and streptomycin (P/S) solution were from EuroClone (Paignton, UK). Neurobasal medium, Leibovitz L15 medium, brain‐derived neurotrophic factor (BDNF), B27 supplement, and MitoSOX™ Red fluorescent stain were purchased from Thermo Fisher Scientific (Waltham, MA, USA). High‐performance thin‐layer chromatography (HPTLC) and silica gel 60 plates were from Merck Millipore (Frankfurter, Germany). Primers and probes were obtained from Eurofins Genomics MWG Operon (Ebersberg, Germany). Mitotracker Red CMXRos was purchased from Cell signaling Technology (Danvers, MA, USA).

#### Antibodies

For immunofluorescence analyses of MNs, the following antibodies were used:

- primary mouse monoclonal antibody anti microtubule‐associated‐protein 2 (MAP2) (Sigma‐Aldrich; Cat# M4403, RRID: AB_477193) and secondary anti‐mouse IgG (H + L) coupled with an Alexa Fluor 488 antibody produced in goat (Sigma‐Aldrich; Cat# SAB4600042, RRID: AB_2532075);
- primary chicken polyclonal antibody anti MAP2 (Abcam, Cambridge, UK; Cat# ab5392, RRID: AB_2138153) and secondary anti‐chicken IgY (H + L) coupled with an Alexa Fluor 568 antibody produced in goat (Sigma‐Aldrich; Cat# SAB4600079, RRID: not available);
- primary rabbit polyclonal antibody anti‐TDP‐43 (Proteintech, Manchester, UK; Cat# 10782‐2‐AP, RRID: not available) and secondary anti‐rabbit IgG (H + L) coupled with an CF™ 568 antibody produced in goat (Sigma‐Aldrich; Cat# SAB4600084, RRID: not available);
- primary mouse monoclonal antibody anti‐SOD1 (g93A mutant) (VWR, Milan, Italy; Cat# MEDMMM‐0070‐P, RRID: not available) and secondary anti‐mouse IgG (H + L) coupled with an Alexa Fluor 488 antibody produced in goat (Sigma‐Aldrich; Cat# SAB4600042, RRID: AB_2532075).

### Cell cultures

#### N2a cell culture

Wild‐type murine N2a cells were cultured as monolayer in DMEM high glucose medium supplemented with 10% heat inactivated (56 °C for 30 min) FBS, 1% l‐glutamine, 1% P/S (penicillin 10 000 U·mL^−1^ and streptomycin 10 mg·mL^−1^), and 1 mm sodium pyruvate, at 37 °C in with 95% air/5% CO_2_. Cells were subcultured to a fresh culture when growth reached the 80–90% confluence (i.e., every 3–4 days).

#### Cell authentication

N2a cells are not listed as a commonly misidentified cell line by the International Cell Line Authentication Committee. N2a cells were bought from Sigma‐Aldrich to which they were supplied by European Collection of Authenticated Cell Cultures (ECACC) (Catalog no. 89121404; Lot no. 13K010, passage +9). N2a were used from passage +10 to passage +15 to conduct experiments reported in the present manuscript. To verify the authentication of employed N2a cells we performed the following tests at the beginning and end of single experimental work.

##### Morphology check by microscope

To identify the state of cells, we checked cellular morphology by phase contrast microscopy (Olympus BX50 microscope; Olympus, Tokyo, Japan). Morphological outcomes of N2a cells confirmed the expected neuronal/ameboid‐like morphology (data not shown).

##### Growth curve analysis

Cell proliferation was evaluated according to MTT method. Briefly, 2.4 mm MTT (4 mg·mL^−1^ in PBS) were added to each well and plates were re‐incubated for 4 h at 37 °C. Medium was carefully discarded and replaced with 2‐propanol : formic acid, 95 : 5 (v:v). Plates were gently agitated prior to read the absorbance at 570 nm with a microplate spectrophotometer (Wallac 1420 VICTOR2TM; Perkin Elmer, Zaventem, Belgium). The growth profile showed a normal growth rate (data not shown).

##### Mycoplasma detection

Mycoplasma infection was evaluated by fluorescent Hoechst staining [31], a fluorescent dye that binds specifically to DNA and that reveals the presence of mycoplasma infections as extracellular particulate or filamentous fluorescence at 100× magnification using NikonEclipse Ni upright microscope. The mycoplasma test has always given negative results (data not shown).

### Primary culture of spinal MNs

The collection of embryos was carried out by trained personnel, in accordance with the National Institutes of Health Guide for the Care and Use of Laboratory Animals and followed current European Union regulations (Directive 2010/63/EU) and was supervised and approved by the local direction of the veterinary services of the Bouches‐du‐Rhône (agreement number B1301310). Rat MNs were cultured as previously described [32, 33]. Briefly, pregnant female rats of 14 days of gestation (Sprague Dawley; Taconic Bioscience, France) were sacrificed using a deep anesthesia with CO_2_ chamber and a cervical dislocation. Then, fetuses were removed from the uterus and immediately placed in ice‐cold L15 Leibovitz medium with a 2% P/S solution and 1% BSA. Spinal cords were treated for 20 min at 37 °C with a trypsin–EDTA solution at a final concentration of 0.05% trypsin and 0.02% EDTA. The dissociation was stopped by the addition of DMEM with 4.5 g·L^−1^ of glucose, containing DNAse I grade II (final concentration 0.5 mg·mL^−1^) and 10% FCS. Cells were mechanically dissociated by three forced passages through the tip of a 10‐mL pipette. Cells were then centrifuged at 515 ***g*** for 10 min at 4 °C. The supernatant was discarded, and the pellet was resuspended in a defined culture medium consisting of Neurobasal medium with a 2% solution of B27 supplement, 2 mmol·L^−1^ of l‐glutamine, 2% of P/S solution, and 10 ng·mL^−1^ of BDNF. Viable cells were counted in a Neubauer cytometer, using the Trypan blue exclusion test. Wild‐type or *SOD1* ^*G93A*^ cells were seeded at a density of 20 000 per well in 96‐well plates precoated with poly‐l‐lysine and were cultured at 37 °C in an air (95%)‐CO_2_ (5%) incubator. The medium was changed every 2 days. The wells of the first lines and columns were not used for culture (to avoid edge effect) and were filled with sterile water.

#### Genotyping of SOD1 Tg embryos

Pregnant female *SOD1* ^*G93A*^ rats (Sprague Dawley; Taconic), of 14 days of gestation, were ordered from Taconic Bioscience. The day of the dissection (from pregnant females at 14 days of gestation), a piece of each embryo brain (~ 3 mm^3^) were placed in a 2‐mL DNAse free tube with a new scalpel. The DNA was extracted with the SYBR Green Extract‐N‐Amp tissue PCR kit (Sigma Aldrich). Briefly, 120 μL of extraction solution were put on each piece of embryo head. Then, they were incubated for 10 min at 23 °C. At the end of the incubation, the heads were incubated for 5 min at 95 °C. Immediately after this last incubation, 100 μL of neutralizing solution was added; each DNA extract was diluted at 1/40 and stored at 4 °C until use. *SOD1* ^*G93A*^ gene presence was determined using genomic fragment with human *SOD1* primers (5′‐CATCAGCCCTAATCCATCTGA‐3′; 5′‐CGCGACTAACAATCAAAGTGA‐3′). The *SOD1* primers were diluted at 3 μm in sterile ultrapure water. Briefly, a mix for PCR was prepared with ultrapure water (4 μL per sample), primer at 3 μm (2 μL per sample) and Master Mix (10 μL per sample). In a PCR 96 well plate, 16 μL of PCR mix were added in each well. 4 μL of each diluted DNA was added according to a plan deposit. The real‐time (RT)‐PCR was run using the CFX96 RT‐PCR system (BioRad, Hercules, CA, US), using the following program: Initial denaturation (95 °C, 20 s)/45 cycles (95 °C, 10 s; 65 °C, 10 s; 72 °C, 30 s)/Melt curve (95 °C, 15 s; 64 °C, 1 min; 90 °C, 30 s; 60 °C, 15 s). The amplification plots and melt curves were analyzed with the Bio‐Rad software. The results for each sample were compared to the negative control (ultrapure water) and to the positive control (DNA from *SOD1* ^*G93A*^ Tg embryos).

### Cells treatments

#### N2a cells treatment

N2a cells were plated at 5 × 10^3^ cells·cm^−2^ and incubated for 24 h to allow cells attachment and recovery in complete medium before treatments. Thus, growth media was withdrawn and N2a cells were pre‐incubated in DMEM high glucose containing 2% inactivated FBS (30 min at 56 °C), 1% l‐glutamine, 1% P/S, and 1 mm sodium pyruvate for 30 min at 37 °C. Sequentially, 50 μm GM1‐OS was added to cells, which were then incubated at 37 °C for 24 h. This dosage was chosen since we have previously demonstrated that it is sufficient to promote neurodifferentiation and neuroprotection in N2a cells by activating the TrkA‐ERK1/2 signaling pathway [34, 35, 36, 37]. To induce an intracellular overload of calcium, following 24 h from GM1‐OS, cells were incubated with DCB (1.5 μm) for 24 h [38]. Control experiments were carried out under the same experimental conditions omitting DCB and/or GM1‐OS addition.

#### MNs treatments

On Day 13 of culture, GM1 and GM1‐OS were dissolved in culture medium and separately administered to MNs at the final concentration of 50 μm [31, 34, 36, 37]. After 1 h after GM1 and GM1‐OS incubation, glutamate was dissolved in medium and added to a final concentration of 5 μm. After 20 min, glutamate was washed out and fresh culture medium supplemented with GM1 or GM1‐OS was added. After 4‐ or 24‐h specific analyses were performed as reported below.

### N2a morphological analysis

Cultured cells, treated or not with GM1‐OS, in the absence or in the presence of DCB, were observed by phase contrast microscopy (20× magnification, Olympus BX50 microscope; Olympus). At least 10 fields from each well were photographed for each experiment.

### Determination of N2a cell viability

Cell viability was determined by Trypan blue exclusion assay previously described [39, 40] after 24 h DCB treatment. At least 20 fields have been evaluated for each sample.

### MNs immunofluorescence assays

For each experimental condition, 30 pictures (representative of all the well's area) per well were automatically taken using ImageXpress® (Molecular Devices, San Jose, CA, US) with 20× magnification, using the same acquisition parameters. From images, analyses were directly and automatically performed by MetaXpress® (Molecular Devices) [41, 42, 43].

#### Survival and neurite networks assessment

Twenty‐four hours after glutamate‐intoxication, MNs were fixed by a cold solution of ethanol (95%) and acetic acid (5%) for 5 min at −20 °C. After permeabilization with 0.1% of saponin, cells were incubated for 2 h at 23 °C with anti‐MAP2 antibody at dilution of 1 : 400 in PBS containing 1% FCS and 0.1% of saponin. The anti‐MAP2 antibody was revealed with Alexa Fluor 488 goat anti‐mouse IgG or with Alexa Fluor 568 anti‐chicken IgG at the dilution 1 : 400 in PBS containing 1% FCS and 0.1% saponin, for 1 h at 23 °C. Survival was evaluated as the number of MAP2‐positive neurons, whereas neurite network was assessed by analyzing the total neurite length of MAP2‐positive neurons in μm. Nuclei were counterstained with the fluorescent dye Hoechst (1 : 1000 in PBS) and utilized as marker of cell number.

#### SOD1 and TDP‐43 analyses

Twenty‐four hours after glutamate‐intoxication, MNs were fixed and permeabilized as described in Survival and neurite networks assessment section. Next, cells were costained with anti‐MAP2 antibody (1 : 400) and antimisfolded SOD1 (G93A) (1 : 400) or antinuclear TDP‐43 (1 : 100) in PBS containing 1% FCS and 0.1% saponin for 2 h at 23 °C. MAP2 signal was revealed with Alexa Fluor 488 goat anti‐mouse IgG or with Alexa Fluor 568 anti‐chicken IgG (1 : 400), misfolded SOD1 with Alexa Fluor 568 goat anti‐mouse (1 : 400) and TDP‐43 with Alexa Fluor 568 goat anti‐rabbit (1 : 400) in PBS containing 1% FCS and 0.1% saponin, for 1 h at 23 °C. Cells were finally incubated with fluorescent dye Hoechst (1 : 1000 in PBS) for staining nuclei to evaluate the cell number. The degree of aggregated SOD1 and the cytoplasmic localization of TDP‐43 were evaluated as the overlap area (μm^2^) of MAP2 and aggregated SOD1 or TDP‐43 signals.

#### Mitochondria density evaluation

Twenty‐four hours after glutamate‐intoxication, the cell culture supernatant was discarded, and the cells were incubated for 45 min at 37 °C with Mitotracker Red CMXRos solubilized at 50 nM in the culture medium. After incubation, the cells were fixed with a cold mixture of 4% paraformaldehyde in PBS (pH = 7.3) for 20 min at 23 °C. The cells were washed twice in PBS, and then permeabilized as described in Survival and neurite networks assessment section. Nonspecific sites were blocked with a solution of PBS containing 0.1% saponin and 1% FCS for 15 min at 23 °C.

Then, the fixed cells were incubated with a mouse monoclonal antibody anti‐MAP2 (1 : 400) in PBS containing 1% FCS and 0.1% saponin for 2 h at 23 °C. MAP2 signal was revealed with Alexa Fluor 488 goat anti‐mouse IgG (1 : 400) in PBS containing 1% FCS and 0.1% saponin, for 1 h at 23 °C. As marker of cell number, nuclei were counterstained with the fluorescent dye Hoechst (1 : 1000 in PBS). Total number of functional mitochondria was evaluated by analyzing Mitotracker Red CMXRos signal overlap in MAP2‐positive neurons area (μm^2^).

#### Mitochondrial ROS analyses

Four hours after glutamate‐intoxication, the cell culture supernatant was discarded, and cells were incubated with MitoSOX™ Red for 10 min at 37 °C. The MitoSOX™ reagent is cell‐penetrant and becomes fluorescent once oxidized by superoxide ($O_{2}^{\cdot -}$). Then, cells were fixed and permeabilized as described in Survival and neurite networks assessment section. Following, cells were incubated with anti‐MAP2 antibody (1 : 400) in PBS containing 1% FCS and 0.1% saponin for 2 h at 23 °C. This antibody was revealed with Alexa Fluor 488 goat anti‐mouse IgG (1 : 400) in PBS containing 1% FCS and 0.1% saponin, for 1 h at 23 °C. Nuclei were stained with the fluorescent dye Hoechst (1 : 1000 in PBS) and used as marker of cell number. By examining the overlapping area (μm^2^) of MitoSOX™ signal in MAP2‐positive neurons, the total number of functioning mitochondria was determined.

### Preparation of ganglioside GM1 and its oligosaccharide

Following a method previously established [44], GM1 ganglioside was isolated from the total ganglioside mixture recovered from fresh pig brains collected at the Galbani company's slaughterhouse (Melzo, Italy). Five grams of ganglioside mixture was dissolved in 500 mL of 50 mm sodium acetate, 1 mm CaCl_2_ buffer, pH = 5.5, that had been prewarmed to 36 °C. Every 12 h, one unit of *V. cholerae* sialidase was added to the solution [45]. The solution was dialyzed at 23 °C for 4 days against 10 L of water that was changed five times daily after being incubated at 36 °C for 2 days while being stirred magnetically. The sialidase‐treated ganglioside mixture was run through a 150 cm × 2 cm silica gel 100 column chromatography system that was pre‐equilibrated and eluted with chloroform/methanol/water, 60 : 35 : 5 by vol. GM1‐containing fractions, identified by thin layer chromatography (TLC), were combined, dried, and subjected to a second column chromatographic (50 cm × 2 cm silica gel 100 column) purification under the aforementioned experimental conditions. Pure GM1‐containing fractions were gathered and dried. The residue was dissolved in 1 L of water and then 4 L of cold acetone were added to precipitate it. The GM1 pellet was centrifuged (15 000 ***g***, 15 min), dried, dissolved in 50 mL of deionized water, and lyophilized to produce 1350 mg of white powder, which was kept at 20 °C. The GM1‐OS was prepared by ozonolysis followed by alkaline degradation of GM1 [46]. Minor changes for the alkaline degradation were introduced. Briefly, GM1 ganglioside was dissolved in the minimum required methanol and slowly saturated with and maintained under ozone at 23 °C for 6 h under continuous stirring. Following the solvent's evaporation under vacuum, triethylamine was added to bring the residue's pH between 10.5 and 11.0. Following solvent evaporation, GM1‐OS was purified by flash chromatography with the following eluent ratios: chloroform/methanol/2‐propanol/water, 60 : 35 : 5 : 5. GM1‐OS was dissolved in sterile ultrapure water and stored at −20 °C. Nuclear Magnetic Resonance (NMR), Mass Spectrometry (MS) and high performance(HP)‐TLC analyses showed a purity over 99% for the prepared oligosaccharide (data not shown, for reference see [31]).

### Statistical analysis

Data are expressed as mean ± SEM and were analyzed for significance by One‐way ANOVA following Tukey's or by Fisher's LSD *post hoc* tests. The analysis was performed with the prism software (GraphPad Software, Inc., La Jolla, CA, USA). The normality distribution was verified using Kolmogorov–Smirnov, D' Agostino & Pearson and Shapiro–Wilk tests. A *P*‐value < 0.05 was considered significant.

## Results

### GM1‐OS protects against DCB‐induced toxicity in N2a cells

The finding that GM1 protected neurons from the toxic effects induced by glutamate and by the calcium ionophore suggests that GM1 is involved in maintaining intracellular calcium homeostasis [23, 24, 25, 26, 27, 47, 48]. Indeed, GM1 influences calcium fluxes by interacting with different players including calcium influx channels and exchangers, and various calcium‐dependent enzymes [21]. Calcium efflux from cytoplasm was found to be modulated by the GM1 association with sodium/calcium exchangers (NCX) in the nuclear envelope [49]. Moreover, we previously demonstrated that GM1 is able to modulate intracellular calcium flux in the murine neuroblastoma cell line (N2a) via direct binding of its oligosaccharide portion to the TrkA receptor on the cell surface leading to the activation of the downstream PLCγ and PKC signaling pathways [37].

**Fig. 1:**
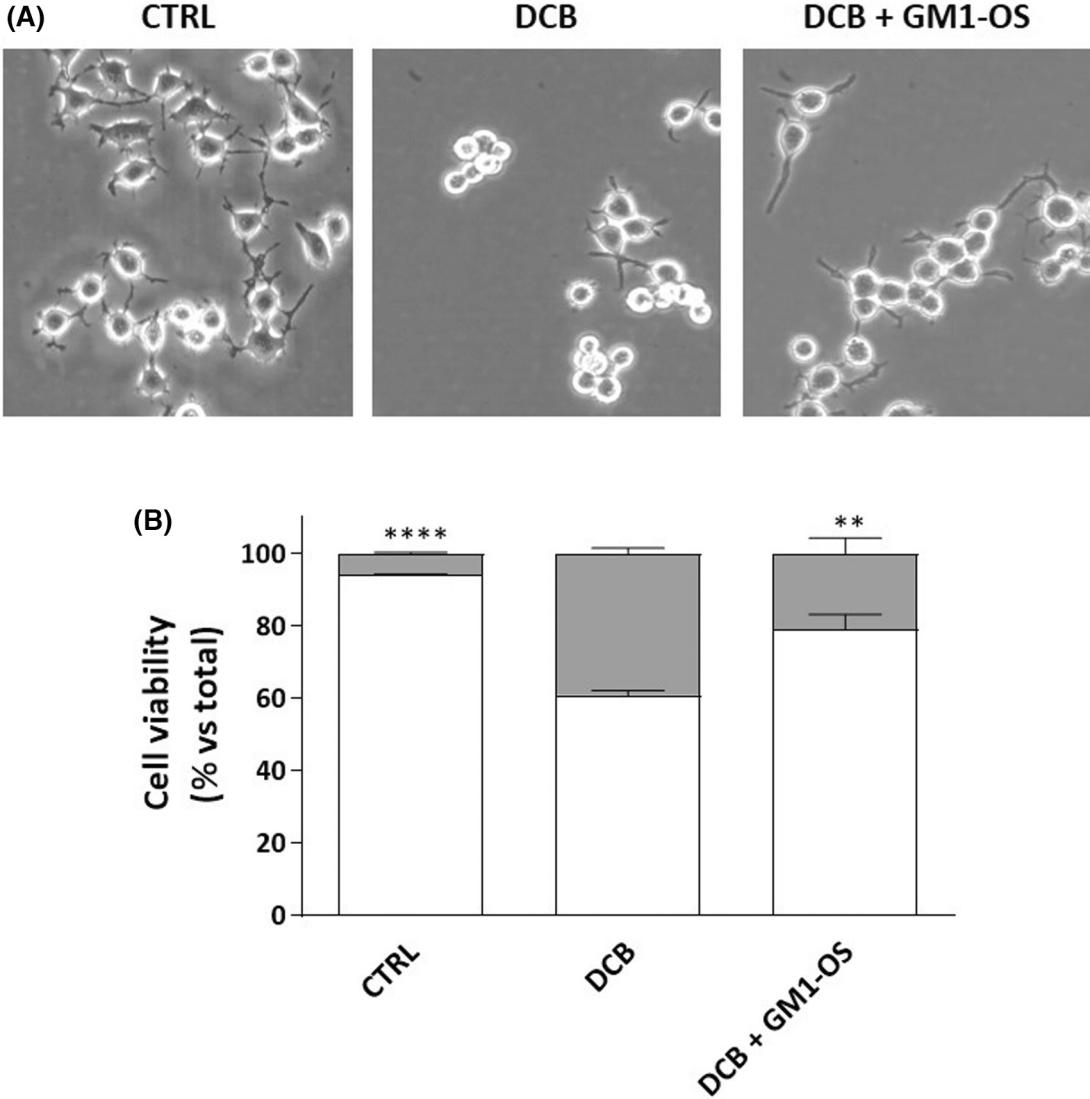
GM1‐OS neuroprotective effect versus DCB treatment in N2a cell line. N2a cells were pre‐treated or not (control, CTRL) with GM1‐OS for 24 h, before DCB exposure. Next, DCB (1.5 μm) or vehicle (CTRL) was added to the culture medium for 24 h. (A) Phase contrast images of N2a cells (20× magnification). Images are representative of three independent experiments (*n* = 3); (B) Trypan Blue viability assay: living cells (white square) and dead cells (gray square). Values are expressed as the percentage mean of living (Trypan blue‐negative) and dead (Trypan blue‐positive) cells (*n* = 3; **P < 0.01; ****P < 0.0001 of living cells by one‐way ANOVA versus DCB, followed by Tukey's *post hoc*).

Thus, to understand whether the GM1‐OS could have a protective role against excitotoxicity, we used a simple model represented by N2a cells exposed to DCB, a potent inhibitor of the sodium/calcium antiporter which induces an intracellular calcium elevation and consequent cell death [38]. As expected, we confirmed that DCB exposure leads to a 40% decrease of cell viability (Fig. 1). Importantly, GM1‐OS pretreatment significantly improved N2a viability. These results highlight that GM1‐OS can exert a protective activity against cell death phenomena induced by calcium overload such as excitotoxicity.

### GM1 preserves WT MNs from excitotoxicity via GM1‐OS

Given the evidence of a potential role of GM1‐OS in counteracting excitotoxicity (Fig. 1), we aimed to assess whether GM1‐OS was directly capable to protect against glutamate‐induced toxicity as GM1 was reported to do [26, 27, 48].

**Fig. 2:**
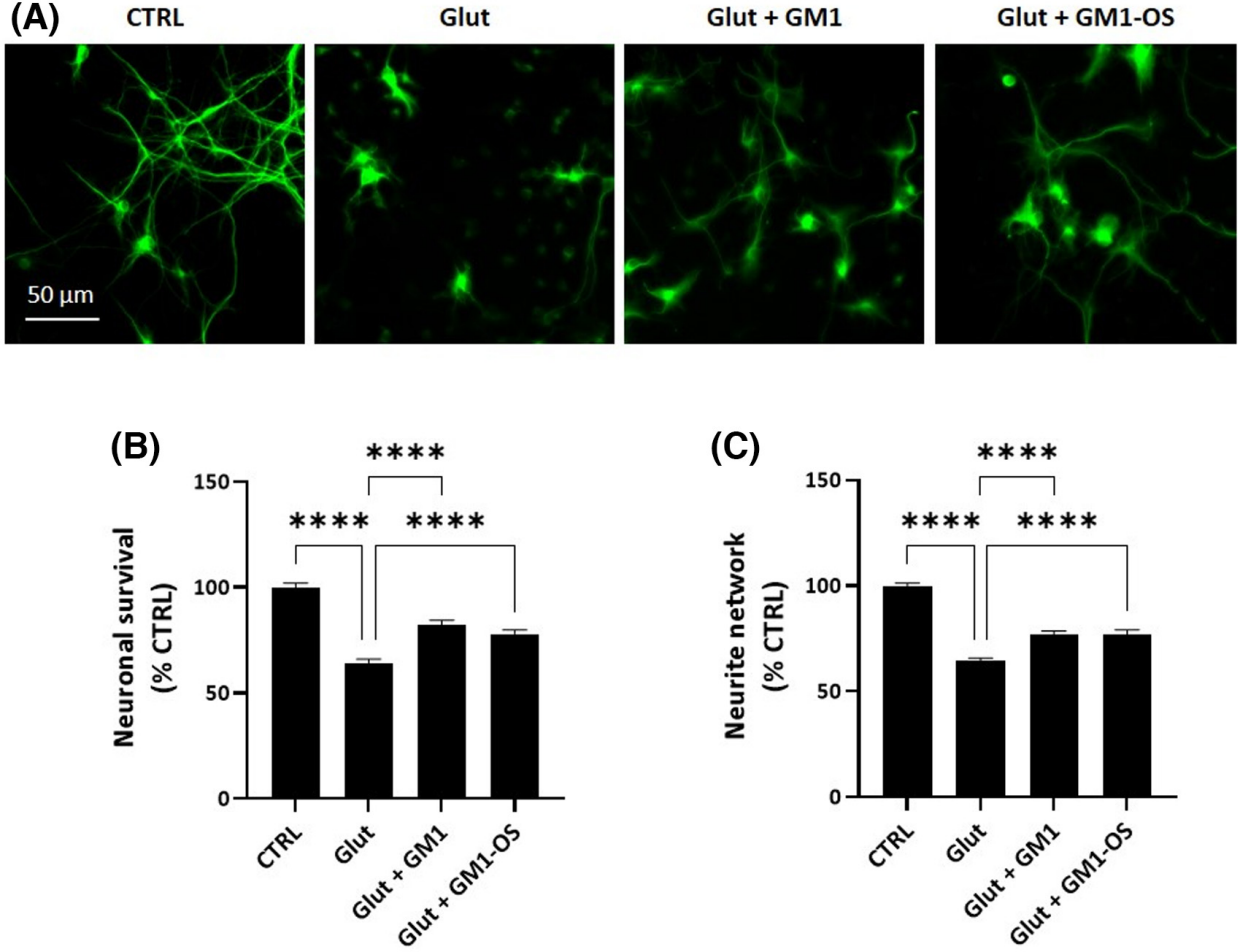
Neuroprotective effects of GM1 and GM1‐OS in a primary culture of rat MNs intoxicated with glutamate. On day 13 of cell culture, primary MNs were pre‐incubated or not (CTRL) with GM1 (50 μm) or GM1‐OS (50 μm) for 1 h, before glutamate exposure. Next, glutamate (5 μm) was added or not (CTRL) to the culture medium. After 20 min, glutamate was washed out and fresh culture medium with GM1 or GM1‐OS was added. After 24 h, MAP2 immunofluorescence was performed as described in the Materials and methods section. (A) Representative immunofluorescence images of MAP2‐positive neurons (20× magnification, scale bar 50 μm); (B) Number of MAP2‐positive neurons, as read‐out of MNs survival; (C) Length of MAP2‐positive neurite in μm, to evaluate the total neurite network of MNs. All values are represented as percentage versus CTRL and expressed as mean ± SEM (*n* = 6; ****P < 0.0001; one‐way ANOVA followed by Fisher's LSD).

To this purpose, we decided to exploit MNs, a population of neurons particularly sensitive to excessive glutamate receptor stimulation. As expected, glutamate exposure dramatically decreased the MNs survival, as shown by a significant decline of MAP2‐positive neurons and neurite network degeneration (Fig. 2). Importantly, MNs pretreated with GM1 and GM1‐OS showed reduced loss of MAP2‐signal (Fig. 2A,B). Moreover, the neurite network of glutamate‐damaged MNs was preserved in presence of GM1 and GM1‐OS (Fig. 2A,C).

These studies demonstrate the protective effect of GM1 against excitotoxicity in glutamate‐MNs, highlighting that this effect can be mediated as well by its oligosaccharide portion alone.

### GM1 reduces SOD1 aggregation and TDP‐43 mislocalization in glutamate‐intoxicated WT MNs via GM1‐OS

A characteristic signature of ASL is the accumulation of ubiquitylated proteinaceous inclusions whose main constituents are predominantly aggregates of SOD1 and TDP‐43 proteins [6]. These proteins can aggregate even in absence of genetic mutations, as a consequence of impaired calcium homeostasis [50, 51, 52].

**Fig. 3:**
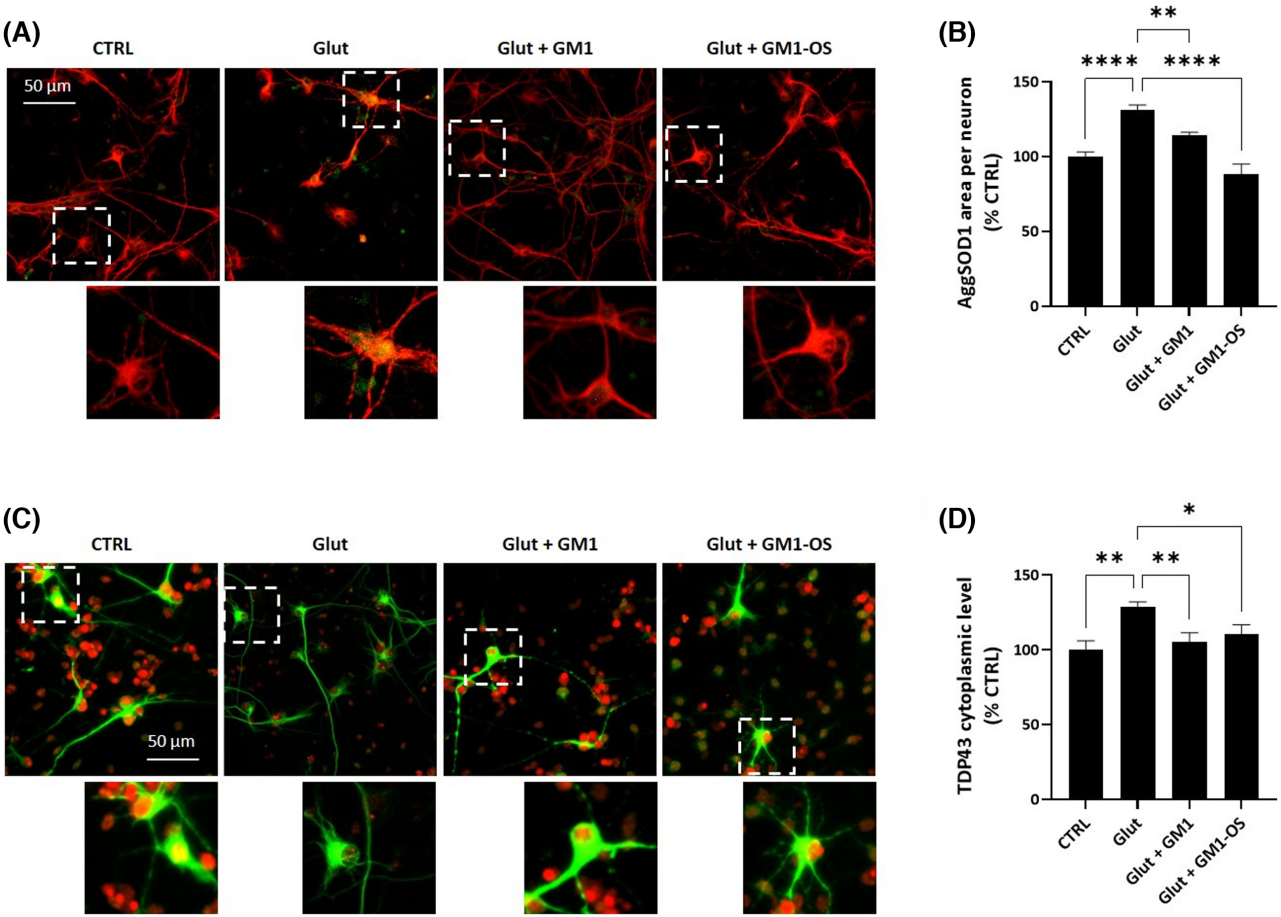
GM1 and GM1‐OS recovery of glutamate‐induced SOD1 aggregation and TDP‐43 mislocalisation in a primary culture of rat MNs. On day 13 of culture, primary MNs were pre‐incubated or not (CTRL) with GM1 (50 μm) or GM1‐OS (50 μm) for 1 h, before glutamate exposure. Next, glutamate (5 μm) was added or not (CTRL) to the culture medium. After 20 min, glutamate was washed out and fresh culture medium with GM1 or GM1‐OS was added. Twenty‐four hours later, SOD1 aggregates and TDP‐43 immunofluorescence were performed as described in the Materials and methods section. (A) Representative immunofluorescence images of MAP2 positive‐neurons co‐stained for SOD1 (20× magnification, scale bar 50 μm) (red MAP2, green SOD1); the dotted line corresponds to the enlarged micrograph region shown; (B) Quantification of SOD1 aggregates in MAP2‐positive neurons (SOD1 aggregates (AggSOD1) in MAP2‐positive area in μm^2^); (C) Representative immunofluorescence images of MAP2 positive neurons co‐stained for TDP‐43 (20× magnification, scale bar 50 μm) (green MAP‐2, Red TDP‐43); the dotted line corresponds to the enlarged micrograph region shown; (D) Quantification of cytoplasmatic TDP‐43 in MAP2‐positive neurons (TDP‐43 staining in MAP2‐positive area in μm^2^). All values are represented as percentage versus CTRL and expressed as mean ± SEM (*n* = 6; *P < 0.05; **P < 0.01; ****P < 0.0001; one‐way ANOVA followed by Fisher's LSD).

To understand whether GM1, along with its oligosaccharide derivative, is able to counteract the pathological aggregation of SOD1 and TDP‐43, we analyzed WT MNs intoxicated with glutamate. In accordance with the literature data [50, 51, 52], glutamate distress boosted SOD1 aggregation, as assessed by a significant increase of the misfolded SOD1 fluorescence signal (Fig. 3A,B). Additionally, upon glutamate administration, we observed TDP‐43 mislocalization from nucleus to cytoplasm, as evaluated by an increase in the TDP‐43 cytoplasmic fluorescence (Fig. 3C,D). Both GM1 and GM1‐OS treatments reduced SOD1 aggregation (Fig. 3A,B) and TDP‐43 mislocalization (Fig. 3C,D) reaching control levels.

These data demonstrate the GM1 efficacy in counteracting SOD1 and TPD‐43 aggregation by its oligosaccharide head.

### GM1 decreases mitochondrial loss and lowers the excess of mitochondrial $O_{2}^{\cdot -}$ in glutamate‐intoxicated WT MNs via GM1‐OS

**Fig. 4:**
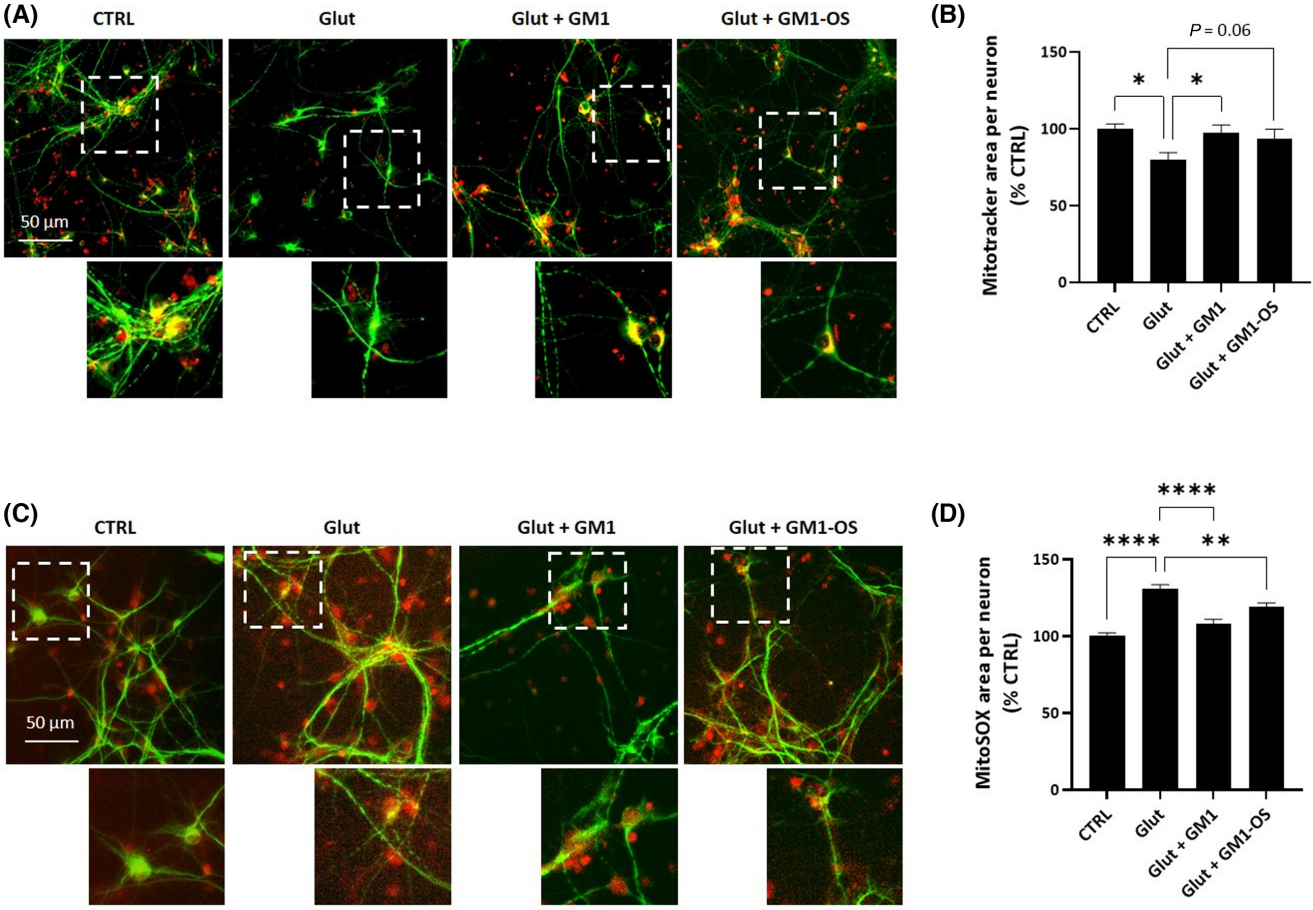
GM1 and GM1‐OS reduction of glutamate‐induced mitochondrial loss and mitochondrial ROS in a primary culture of rat MNs. On day 13 of culture, MNs were pre‐incubated or not (CTRL) with GM1 (50 μm) or GM1‐OS (50 μm) for 1 h, before glutamate exposure. Next, glutamate (5 μm) was added or not (CTRL) to the culture medium. After 20 min, glutamate was washed out and fresh culture medium with GM1 or GM1‐OS was added. After 4 h, mitochondrial $O_{2}^{\cdot -}$ levels were evaluated with MitoSOX Red and after 24 h, mitochondrial network was evaluated using Mitrotracker Red CMXRos, as described in the Materials and methods section. (A) Representative immunofluorescence images of MNs co‐stained with anti‐MAP2 and Mitrotracker Red CMXRos dye (20× magnification, scale bar 50 μm) (green MAP2, red Mitotracker); the dotted line corresponds to the enlarged micrograph region shown; (B) Quantification of Mitrotracker Red CMXRos overlapping with MAP2‐positive area in μm^2^; (C) Representative immunofluorescence images of MNs co‐stained with anti‐MAP2 and MitoSOX Red dye (20× magnification, scale bar 50 μm) (green MAP‐2, red MitoSOX); the dotted line corresponds to the enlarged micrograph region shown; (D) Quantification of MitoSOX Red signal overlapping with MAP2‐positive area in μm^2^. All values are represented as percentage versus CTRL and expressed as mean ± SEM (*n* = 6; *P < 0.05; **P < 0.01; ****P < 0.0001; one‐way ANOVA followed by Fisher's LSD).

The glutamate‐cytotoxicity is linked to mitochondrial dysfunction deriving from increased oxygen consumption and consequent boosting of ROS levels, altered mitochondrial polarization, imbalanced mitochondrial dynamics, and mitochondrial calcium handling [3, 53]. Considering the capability of GM1 and GM1‐OS to modulate mitochondria function and to scavenge oxidative stress [31, 36, 48, 54, 55], we decided to evaluate mitochondria status in MNs challenged with exogenous glutamate (Fig. 4).

The network of functional mitochondria was identified with Mitotracker Red CMXRos dye [56], after 24 h of exposure to glutamate (Fig. 4A). As shown in Fig. 4B, glutamate induced a 20% reduction of mitochondrial mass in MAP2‐positive neurons that was significantly recovered by GM1, but not by its oligosaccharide which induced only a partial but not significant recovery (*P* = 0.06).

Next, we evaluated mitochondrial $O_{2}^{\cdot -}$ using the specific MitoSOX Red dye [36, 41, 57] 4 h after glutamate administration (Fig. 4C). As shown in Fig. 4D, glutamate significantly increased $O_{2}^{\cdot -}$ levels, while both GM1 and GM1‐OS treatments lowered mitochondrial $O_{2}^{\cdot -}$ overload in glutamate‐intoxicated MNs. Four hours after glutamate administration neither the decrease in MNs viability or the degeneration of neurite network were found, indicating that the ROS increase strictly depends on impaired mitochondria homeostasis (Fig. S1).

These results suggest that GM1 neuroprotective properties against glutamate excitotoxicity may involve the modulation of mitochondrial dynamics and activity through its oligosaccharide.

### GM1 preserves *SOD1* ^*G93A*^ MNs from excitotoxicity via GM1‐OS

Considering the protective effect of GM1 and GM1‐OS against excitotoxicity induced by glutamate in WT MNs, we decided to verify their activity in a genetic model of ALS, a neurodegenerative disease where excitotoxicity‐derived damage strongly contributes to neuronal degeneration. Mutation in *SOD1* gene are the most frequently observed in ALS disease [58], and the G93A substitution (glycine 93 converted to alanine) in *SOD1* has been extensively studied because it was the first mutation discovered to cause MNs degeneration in a transgenic mouse model [59]. Interestingly, MNs in mixed spinal cord cultures from *SOD1* ^*G93A*^ mice were found to be more susceptible to glutamate intoxication as compared to the WT counterpart [60].

Thus, to model ALS *in vitro*, we generated MNs from *SOD1* ^*G93A*^ rats and exposed them to glutamate.

**Fig. 5:**
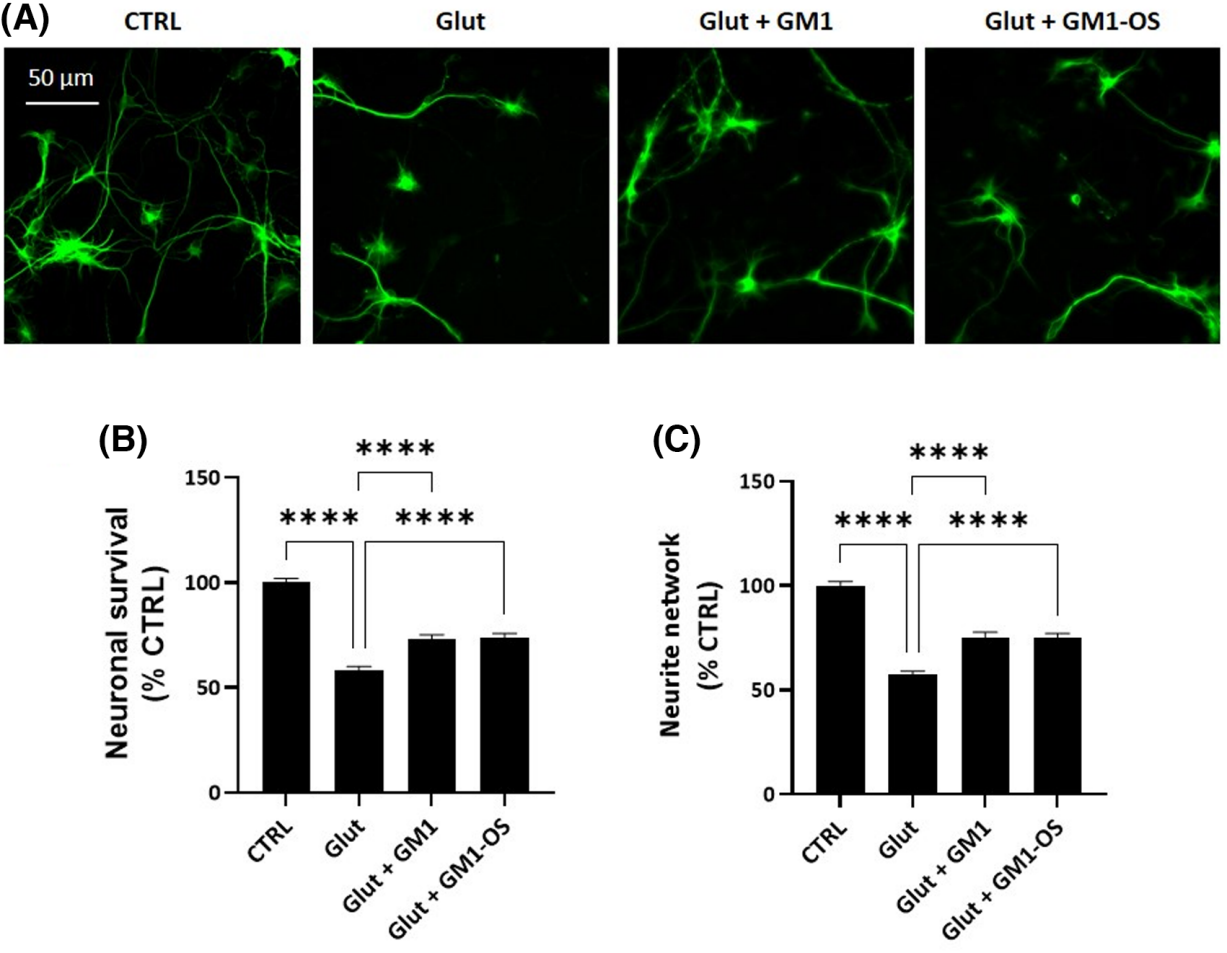
Neuroprotective effects of GM1 and GM1‐OS in a primary culture of rat *SOD1* ^*G93A*^ MNs injured with glutamate. On day 13 of culture, primary MNs were pre‐incubated or not (CTRL) with GM1 (50 μm) or GM1‐OS (50 μm) for 1 h, before glutamate exposure. Next, glutamate (5 μm) was added or not (CTRL) to the culture medium. After 20 min, glutamate was washed out and fresh culture medium with GM1 or GM1‐OS was added. After 24 h, MAP2 immunofluorescence was performed as described in the Materials and methods section. (A) Representative immunofluorescence images of MAP2‐positive neurons (20× magnification, scale bar 50 μm); (B) Number of MAP2‐positive neurons, as read‐out of MNs survival; (C) Length of MAP2‐positive neurite in μm, to evaluate the total neurite network of MNs. All values are represented as percentage versus CTRL and expressed as mean ± SEM (*n* = 6; ****P < 0.0001; one‐way ANOVA followed by Fisher's LSD).

Glutamate exposure exerted a toxic effect in *SOD1* ^*G93A*^ MNs, as showed by around 40% survival decrease and neurite network degeneration (Fig. 5). Importantly, GM1 and GM1‐OS significantly ameliorated *SOD1* ^*G93A*^ MNs phenotype by increasing the number of MAP2‐positive MNs (Fig. 5A,B) and counteracting the fragmentation of the neurite network (Fig. 5A,C).

These data highlight the GM1 protective effect against excitotoxicity in a genetic MNs model of ALS and that this function is mediated by its oligosacchairide.

### GM1 reduces SOD1 aggregation and TDP‐43 mislocalization in glutamate‐injured *SOD1* ^*G93A*^ MNs via GM1‐OS

*SOD1* mutations induce the aberrant folding and subsequent aggregation of SOD1 protein. In addition, mutant SOD1^G93A^ directly interacts with TDP‐43 affecting its solubility and resulting in the redistribution of TDP‐43 from the nucleus to the cytoplasm in an aggregation‐prone state [6, 50, 61].

**Fig. 6:**
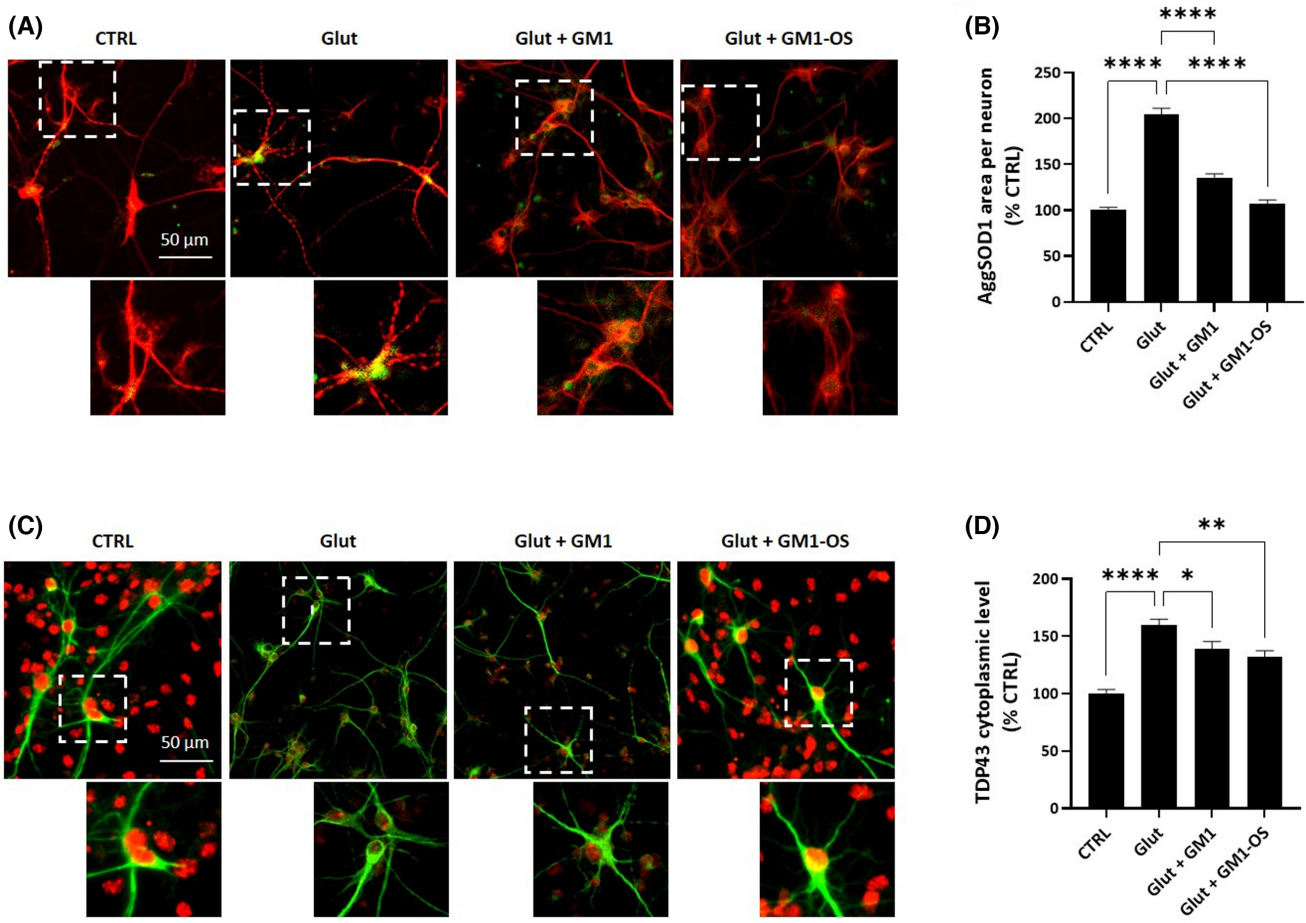
GM1 and GM1‐OS recovery of glutamate‐induced SOD1 aggregation and TDP‐43 mislocalisation in a primary culture of rat *SOD1* ^*G93A*^ MNs. On day 13 of culture, primary MNs were pre‐incubated or not (CTRL) with GM1 (50 μm) or GM1‐OS (50 μm) for 1 h, before glutamate exposure. Next, glutamate (5 μm) was added or not (CTRL) to the culture medium. After 20 min, glutamate was washed out and fresh culture medium with GM1 or GM1‐OS was added. After 24 h, SOD1 and TDP‐43 immunofluorescences were performed as described in Materials and methods section. (A) Representative immunofluorescence images of MAP2‐positive neurons co‐stained for SOD1 (20× magnification, scale bar 50 μm) (red MAP2, green SOD1); the dotted line corresponds to the enlarged micrograph region shown; (B) Quantification of SOD1 aggregates in MAP2‐positive neurons (SOD1 aggregates (AggSOD1) in MAP2‐positive area in μm^2^); (C) Representative immunofluorescence images of MAP‐2 positive neurons co‐stained for TDP‐43 (20× magnification, scale bar 50 μm) (green MAP2, red TDP‐43); the dotted line corresponds to the enlarged micrograph region shown; (D) Quantification of cytoplasmatic TDP‐43 in MAP2‐positive neurons (TDP‐43 staining in MAP2‐positive area in μm^2^). All values are represented as percentage versus CTRL and expressed as mean ± SEM (*n* = 6; *P < 0.05; **P < 0.01; ****P < 0.0001; one‐way ANOVA followed by Fisher's LSD).

Thus, we decided to test whether GM1 and its oligosaccharide were able to inhibit SOD1 aggregation and TDP‐43 mislocalization by exploiting the *SOD1* ^*G93A*^ MNs intoxicated with glutamate. After 24 h from 20‐min glutamate addition, we found that excitotoxin exposure dramatically increased the SOD1 aggregate levels (Fig. 6A,B). Interestingly, GM1 and GM1‐OS pretreatment strongly decreased the aggregation of mutant SOD1^G93A^, reaching control levels in GM1‐OS treated‐ *SOD1* ^*G93A*^ MNs (Fig. 6A,B). Moreover, the analysis of TDP‐43 subcellular distribution revealed an increased expression in the cytoplasm upon glutamate challenge that was significantly mitigated by GM1 and GM1‐OS (Fig. 6C,D).

These results indicate that GM1, via GM1‐OS, counteracts the toxic accumulation and the consequent aggregation of misfolded proteins typically found in SOD1‐dependent ALS.

### GM1 decreases mitochondrial loss and lowers the excess of mitochondrial $O_{2}^{\cdot -}$ in glutamate‐intoxicated *SOD1* ^*G93A*^ MNs via GM1‐OS

Beyond the mitochondrial dysfunction induced by glutamate excitotoxicity, *SOD1* mutations are directly involved in mitochondria distress. Indeed, SOD1 has a predominant localization in the cytoplasm but it is also physiologically localized within mitochondria where it acts as antioxidant by dismutating $O_{2}^{\cdot -}$ to molecular oxygen (O_2_) and hydrogen peroxide (H_2_O_2_) [62]. At mitochondria level, *SOD1* ^*G93A*^ mutant animal models display an increased dismutase activity and ROS production, finally leading to mitochondrial impairment, that is further exacerbated upon cellular stress [7, 60, 62, 63]. Accordingly, *SOD1* mutant MNs displayed an increased vulnerability to glutamate toxicity that is linked to increased ROS generation, elevation of intracellular calcium, and mitochondrial dysfunction [60].

**Fig. 7:**
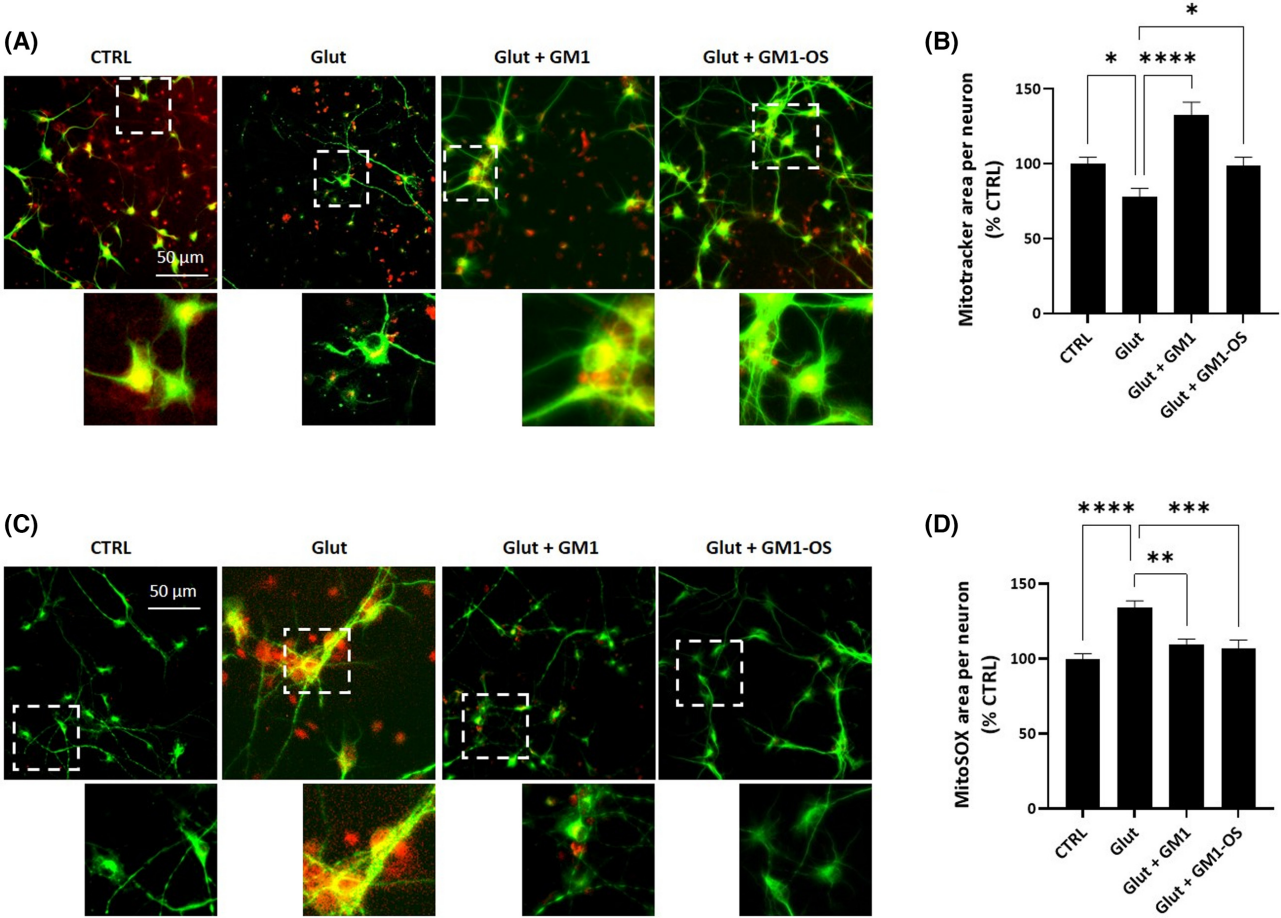
GM1 and GM1‐OS reduction of glutamate‐induced mitochondrial loss and mitochondrial ROS in a primary culture of rat *SOD1* ^*G93A*^ MNs. On day 13 of culture, MNs were pre‐incubated or not (CTRL) with GM1 (50 μm) or GM1‐OS (50 μm) for 1 h, before glutamate exposure. Next, glutamate (5 μm) was added or not (CTRL) to the culture medium. After 20 min, glutamate was washed out and fresh culture medium with GM1 or GM1‐OS was added. After 4 h, mitochondrial $O_{2}^{\cdot -}$ was evaluated with MitoSOX Red and after 24 h, mitochondrial network was evaluated with Mitrotracker Red CMXRos as described in the Materials and methods section. (A) Representative immunofluorescence images of MNs co‐stained with anti‐MAP2 and Mitrotracker Red CMXRos dye (20× magnification, scale bar 50 μm) (green MAP2, red Mitrotracker); the dotted line corresponds to the enlarged micrograph region shown; (B) Quantification of Mitrotracker Red CMXRos signal overlapping MAP2‐positive area in μm^2^; (C) Representative immunofluorescence images of MNs co‐stained with anti‐MAP2 and MitoSOX Red dye (20× magnification, scale bar 50 μm) (green MAP2, red MitoSOX); the dotted line corresponds to the enlarged micrograph region shown; (D) Quantification of MitoSOX Red signal overlapping MAP2‐positive area in μm^2^. All values are represented as percentage versus CTRL and expressed as mean ± SEM (*n* = 6; *P < 0.05; **P < 0.01; ***P < 0.001; ****P < 0.0001; one‐way ANOVA followed by Fisher's LSD).

Thus, we decided to evaluate whether mitochondrial mass and mitochondria ROS production could be recovered by GM1 and GM1‐OS in glutamate‐injured MNs generated from *SOD1* ^*G93A*^ rat embryos. As expected, the administration of excitotoxin induced a reduction of mitochondrial density as assessed by the Mitotracker signal in MAP2‐positive *SOD1* ^*G93A*^ MNs (Fig. 7A,B). Interestingly, the treatment with GM1 and GM1‐OS significantly counteracted the loss of mitochondria mass (Fig. 7A,B). Furthermore, glutamate significantly increased the mitochondrial $O_{2}^{\cdot -}$ levels, 4 h after administration. Importantly, GM1 and GM1‐OS treatments lowered mitochondrial $O_{2}^{\cdot -}$ overload in glutamate *SOD1* ^*G93A*^ MNs 4 h of postglutamate‐injury (Fig. 7C,D).

These results suggest that the GM1 protective properties against excitotoxicity in *SOD1* ^*G93A*^ MNs could be linked to its capability to reduce oxidative stress acting at a mitochondrial level via its oligosaccharide head.

## Discussion

Gangliosides, sialic acid‐containing glycosphingolipids abundant in neuronal membranes, are required for the development and maintenance of the CNS [14, 15, 16, 17]. Accordingly, altered ganglioside metabolism is reported in severe neurodevelopmental and neurodegenerative disorders [64]. For example, the loss of GM2/GD2 synthase, resultant in a deficit of GM2, GD2, GM1 and all other gangliotetraose gangliosides, was reported to be responsible for increased cell death upon glutamate intoxication. The enhanced susceptibility to excitotoxicity seems to be specifically linked to GM1 deficiency since supplementation with GM1 or GM1‐derivative LIGA20 was able to rescue GM2/GD2 synthase knock‐out cells facilitating the restoration of calcium homeostasis [26, 27]. This evidence is strongly sustained by previous evidence highlighting the capability of GM1 to avoid toxic effects of glutamate overload [24, 25, 47]. In contrast, a neuroprotective effect of GD3 or GM3 in the brain has not been documented, aligning with the observation that these ganglioside species exhibit significant expression during developmental stages but relatively minimal in adult life [64].

By analyzing the single molecular components of the ganglioside GM1, we recently discovered that the bioactive portion resides within its oligosaccharide (GM1‐OS). Conversely, even smaller structural elements of the GM1 oligosaccharide, such as the GM2 oligosaccharide II^3^Neu5Ac‐Gg_3_, the GM3 oligosaccharide II^3^Neu5Ac‐Lac (sialyl lactose), the galactose or the sialic acid failed to elicit neuronal functions [34]. GM1‐OS, lacking the hydrophobic tail of ceramide, becomes a soluble and hydrophilic molecule: without entering the cell, it remains in the extracellular environment [18, 34, 35] and interacts directly with the neurotrophin receptor TrkA by mediating the protective and reparative signaling typical of GM1 [18, 34, 65]. On the contrary, GM1‐OS, lacking the amphiphilicity of ganglioside, has the advantage of efficiently crossing the blood brain barrier, showing a 20‐fold higher crossing speed than GM1 and a time‐ and concentration‐dependent paracellular transport [66]. Moreover, after crossing the barrier, GM1‐OS remains intact and retains its neurotrophic properties [66]. Therefore, GM1 exerts its bioactivity through its hydrophilic head, which protrudes into the extracellular environment and then acts on the cell surface by interacting with proteins residing on the plasma membrane. Specifically on the cells surface, by directly binding NGF TrkA receptor activates the ERK1/2 downstream cascade, triggering neuronal differentiation, maturation, and protection [30, 34, 35, 36]. As demonstrated by bioinformatics analyses, the saccharide sequence of GM1‐OS is crucial for the stabilization of the TrkA/NGF complex [34]. An array of molecular processes was shown to be induced by GM1‐OS in a TrkA‐dependent manner, including antioxidant mechanisms, mitochondrial bioenergetics [31], and an anti‐inflammatory response [36]. Accordingly, GM1‐OS was found to be able to counteract neurodegeneration *in vitro* and *in vivo* in Parkinson's disease models [36, 41, 67, 68].

Among other cell events, we demonstrated that GM1‐OS is involved in the regulation of intracellular calcium fluxes in neuroblastoma cells [37], similarly to GM1 [21, 49].

Thus, here, based on the preliminary data showing that GM1‐OS sustains the survival of N2a cells exposed to excessive calcium increase induced by the DCB inhibition of sodium/calcium exchanger current (Fig. 1), we investigated the specific role of GM1, via its oligosaccharide, in protecting against excitotoxicity. We exploited glutamate‐injured MNs since this neuronal population is particularly vulnerable to glutamate toxicity and this condition is further exacerbated in presence of ALS‐causing mutations [2, 60]. By immunofluorescence‐based assays, we showed that the decreased viability and degeneration of neurite network induced by glutamate exposure were significantly recovered by GM1 in both WT and *SOD1* ^*G93A*^ MNs (Figs 2 and 5), confirming previous research that highlighted the protective activity of gangliosides in ALS models [10]. GM1 and GM1‐OS efficacies in counteracting glutamate excitotoxicity in MNs were comparable (Figs 2 and 5), indicating that the oligosaccharide head of GM1, separated from the ceramide tail, was sufficient to exert neuronal protective functions. These data corroborate that GM1‐OS owns the bioactive properties of the ganglioside GM1.

Cellular stress has been demonstrated to induce protein aggregation forming toxic intracellular inclusions, which may exacerbate the damage prompted by excitotoxicity. Among them, aggregates of WT SOD1 have been reported to trigger MNs death via increasing oxidative stress and mitochondria dysfunction [50]. In addition, altered calcium flux has been found to stimulate the cytoplasmic TDP‐43 aggregation by boosting calpain‐dependent TDP‐43 cleavage [51, 52]. Accumulation of TDP‐43 aggregates determines an impairment on nuclear activities in which TDP‐43 is normally involved (i.e., mRNA maturation and transport, repair of genome double‐stranded breaks) as well as alteration of the cytoplasmic compartment (i.e., stress granule modulation, mRNA stability and trafficking, translational and microRNA regulation), indicating that TDP‐43 dysregulation causes systemic cellular dysfunction [69]. Furthermore, mutations in *SOD1* promote the formation of cytoplasmic inclusions, whose main components are misfolded SOD1 and TDP‐43 proteins, and this event is exacerbated by glutamate overstimulation [6, 50, 61].

Accordingly, we found that acute glutamate exposure increases SOD1/SOD1^G93A^ aggregation and cytoplasmic mislocalization of TDP‐43 (Figs 3 and 6) [51]. Interestingly, GM1 supplementation significantly ameliorated the phenotype both in WT and in *SOD1* ^*G93A*^ MNs intoxicated with glutamate (Figs 3 and 6). The same effect was observed by the administration of GM1‐OS alone, indicating that the oligosaccharide portion of GM1 was sufficient in preventing the glutamate induced SOD1/SOD1^G93A^ and TDP‐43 abnormalities. This evidence well correlates with studies demonstrating the role of GM1 in preventing misfolding and aggregation of alpha‐synuclein, the main component of intracellular aggregates characteristic of Parkinson's disease [18]. Remarkably, GM1 capability to inhibit alpha‐synuclein fibrillation has been proved to be mediated by its oligosaccharide head [68, 70, 71].

Additionally, mitochondrial impairment represents a key feature in ALS pathology [72]. Mutant SOD1 localizes at mitochondria and induces the alteration of mitochondrial homeostasis, increasing ROS production that results higher upon excitotoxic stimuli [7, 60, 62, 63]. In both MNs experimental models, glutamate led to accumulation of mitochondrial $O_{2}^{\cdot -}$, which was significantly reduced by GM1 via its oligosaccharide (Figs 4 and 7).

Additionally, the glutamate administration induced a loss in mitochondria mass both in WT and *SOD1* ^*G93A*^ MNs, which was recovered by GM1 in both models and by its oligosaccharide only in *SOD1* ^*G93A*^ MNs (Figs 4 and 7). Interestingly, Kumari *et al*. demonstrated that glutamate at toxic concentrations affected mitochondrial function inducing mitochondrial autophagy (mitophagy). Additionally, the authors demonstrated that selenium supplementation increased cell viability, reduced autophagy activation, and pDrp1‐LC3 overlapping [53]. Based on these observations, we can speculate that reduced mitochondrial mass upon glutamate exposure might involve the activation of mitophagy and that GM1 and GM1‐OS can support mitochondria integrity, thus avoiding their elimination. Accordingly, GM1 has been described as a modulator of autophagic processes [73, 74]. Interestingly, the capability of GM1 and GM1‐OS to preserve mitochondria and avoid ROS accumulation is in line with data reported in literature. GM1, via GM1‐OS, has been demonstrated to counteract the toxicity of 1‐methyl‐4‐phenyl‐1,2,3,6‐tetrahydropyridine hydrocholoride (MPTP), a neurotoxin able to induce neurodegeneration by inhibiting mitochondrial complex I [18, 36, 41]. Additionally, we specifically found that GM1‐OS stimulated mitochondria biogenesis, boosted mitochondria function and reduced mitochondria ROS levels in neuronal cells [31, 41]. Importantly, GM1‐OS potentiated mitochondria function by boosting mitochondrial complex I and II activities in a N2a model of mitochondrial dysfunction [31].

The present study demonstrates the *in vitro* GM1 therapeutic potential in models of Amyotrophic Lateral Sclerosis providing evidence of its protective and restorative ability through its oligosaccharide chain in counteracting glutamate‐induced neurotoxicity in WT and *SOD1* ^*G93A*^ MNs by modulating mitochondrial homeostasis. However additional experimental work is required to fully understand the molecular mechanisms underlying GM1 action in ALS and to specifically verify whether it depends on the modulation of neurotrophins' receptors at plasma membrane.

## Conclusions

Our study demonstrates that GM1 is able to protect WT and ALS MNs against glutamate‐mediated excitotoxicity. Importantly, its function strictly depends on its oligosaccharide association with the cell surface. Indeed, the use GM1‐OS, which is not internalized by cells, faithfully replicates GM1‐induced neuroprotection [30]. In this context, GM1‐OS retains all the beneficial properties of the entire ganglioside but loses its amphiphilicity, enabling it to effectively penetrate the central nervous system [66, 67]. Its biological activity and its ability to penetrate in the brain makes GM1‐OS a promising drug candidate for neurological disorders [30]. Additionally, the capability to counteract the toxic effect of glutamate and oxidative stress seems to be particularly interesting in the ALS context. Indeed, riluzole, a glutamatergic neurotransmitter inhibitor, is currently used to manage ALS and has been demonstrated to extend the life span of patients up to 2 years. Additionally, Edaverone was recently approved for treatment of ALS and its therapeutic activity is likely to be linked to its antioxidant properties [75]. The GM1 oligosaccharide seems to possess all the characteristics to counteract the multifactorial aspects underlying neurodegenerative diseases, and, in the specific case of ALS, to counteract glutamate‐mediated excitotoxicity, mitochondrial dysfunction, ROS overproduction and protein misfolding, thus ensuring neuronal survival.

## Conflict of interest

The authors declare no conflict of interest.

### Peer review

The peer review history for this article is available at https://www.webofscience.com/api/gateway/wos/peer‐review/10.1002/2211‐5463.13727.

## Author contributions

GL, EDB, EVC, MF, AH, NC, MS, and EC were involved in conceptualization, methodology, investigation, analysis, and draft of the manuscript; GL, MF, and EC were involved in supervision, conceptualization, draft and revision of the manuscript; LaM and MGC were involved in GM1‐OS chemical synthesis; GL, EDB, EVC, MF, AH, NC, NL, MA, SS, LaM, LuM, MS, and EC were involved in the revision of the manuscript; all authors have read and agreed to the published version of the manuscript.

## Supporting information


**Fig. S1.** Neuroprotective effects of GM1 and GM1‐OS in a primary culture of WT or *SOD1* ^*G93A*^ rat MNs injured with glutamate. On Day 13 of culture, primary MNs were pre‐incubated or not (CTRL) with GM1 (50 μm) or GM1‐OS (50 μM) for 1 h, before glutamate exposure. Next, glutamate (5 μM) was added or not (CTRL) to the culture medium. After 20 min, glutamate was washed out and fresh culture medium with GM1 or GM1‐OS was added. After 4 h, MAP2 immunofluorescence staining was performed as described in the Methods section. (a) Number of MAP2‐positive neurons, as read‐out of MNs survival in WT MNs; (b) Length of MAP2‐positive neurite of WT MNs in μm, to evaluate the total neurite network of MNs; (c) Number of MAP2‐positive neurons, as read‐out of MNs survival of *SOD1* ^*G93A*^ MNs; (d) Length of MAP2‐positive neurite in μm, to evaluate the total neurite network of *SOD1* ^*G93A*^ MNs. All values are represented as % versus CTRL and expressed as mean ± SEM (*n* = 6, * *p* < 0.05; one‐way ANOVA followed by Fisher's LSD).

## Data Availability

The data presented in this study are available upon reasonable request to the corresponding author.
