# Supplementary material for: GM1 ganglioside exerts protective effects against glutamate‐excitotoxicity via its oligosaccharide in wild‐type and amyotrophic lateral sclerosis motor neurons
Source: FEBS Open Bio. 2023 Nov 15;13(12):2324–41. doi: 10.1002/2211-5463.13727 (PMC10699117; doi:10.1002/2211-5463.13727)
Supplement: Supplementary file 1 — Fig. S1. Neuroprotective effects of GM1 and GM1‐OS in a primary culture of WT or SOD1 G93A rat MNs injured with glutamate. On Day 13 of culture, primary MNs were pre‐incubated or not (CTRL) with GM1 (50 μm) or GM1‐OS (50 μM) for 1 h, before glutamate exposure. Next, glutamate (5 μM) was added or not (CTRL) to the culture medium. After 20 min, glutamate was washed out and fresh culture medium with GM1 or GM1‐OS was added. After 4 h, MAP2 immunofluorescence staining was performed as described in the Methods section. (a) Number of MAP2‐positive neurons, as read‐out of MNs survival in WT MNs; (b) Length of MAP2‐positive neurite of WT MNs in μm, to evaluate the total neurite network of MNs; (c) Number of MAP2‐positive neurons, as read‐out of MNs survival of SOD1 G93A MNs; (d) Length of MAP2‐positive neurite in μm, to evaluate the total neurite network of SOD1 G93A MNs. All values are represented as % versus CTRL and expressed as mean ± SEM (n = 6, * p < 0.05; one‐way ANOVA followed by Fisher's LSD). [file FEB4-13-2324-s001.docx]

***Supporting Information***

**GM1 ganglioside exerts protective effects against glutamate-excitotoxicity via its oligosaccharide in wild-type and amyotrophic lateral sclerosis motor neurons**

Giulia Lunghi^1^ꭞ, Erika Di Biase^1^ꭞ, Emma Veronica Carsana^1^, Alexandre Henriques^2^, Noelle Callizot^2^, Laura Mauri^1^, Maria Grazia Ciampa^1^, Luigi Mari^3^, Nicoletta Loberto^1^, Massimo Aureli^1^, Sandro Sonnino^1^, Michael Spedding^4^, Elena Chiricozzi^1^* and Maria Fazzari^1^

^1^Department of Medical Biotechnology and Translational Medicine, University of Milano, Segrate, Italy

^2^Neuro-sys, Gardanne, France

^3^Department of Immunology, St. Jude Children's Research Hospital, Memphis, TN 38105, USA

^4^Spedding Research Solutions, Le Vésinet, France

ꭞCo-First Authorship

*Correspondence to:

Elena Chiricozzi, Professor

Via Fratelli Cervi 93, 20054 Segrate (MI), Italy

Phone: +39 0250330364

elena.chiricozzi@unimi.it

**Running title:**

GM1 against excitotoxity

**ORCID ID**Elena Chiricozzi 0000-0001-7431-9207

**Supplementary Figure S1**

**
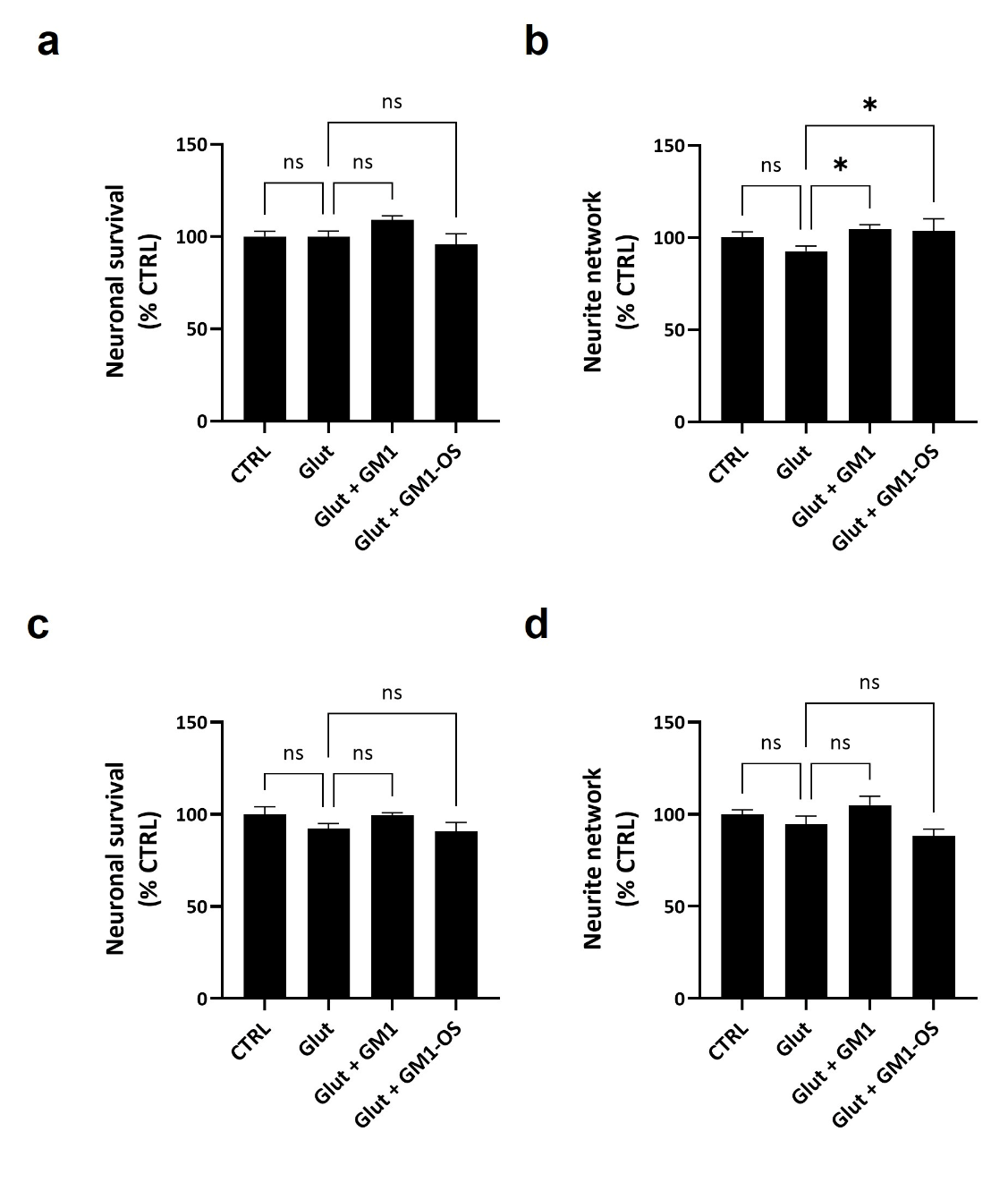
**

**Fig. S1** Neuroprotective effects of GM1 and GM1-OS in a primary culture of WT or *SOD1^G93A^* rat MNs injured with glutamate. On day 13 of culture, primary MNs were pre-incubated or not (CTRL) with GM1 (50 μM) or GM1-OS (50 μM) for 1 h, before glutamate exposure. Next, glutamate (5 μM) was added or not (CTRL) to the culture medium. After 20 min, glutamate was washed out and fresh culture medium with GM1 or GM1-OS was added. After 4 h, MAP2 immunofluorescence staining was performed as described in the Methods section. (**a**) Number of MAP2-positive neurons, as read-out of MNs survival in WT MNs; (**b**) Length of MAP2-positive neurite of WT MNs in µm, to evaluate the total neurite network of MNs; (**c**) Number of MAP2-positive neurons, as read-out of MNs survival of *SOD1^G93A^* MNs; (**d**) Length of MAP2-positive neurite in µm, to evaluate the total neurite network of *SOD1^G93A^* MNs. All values are represented as % versus CTRL and expressed as mean ± SEM (n = 6, * p < 0.05; one-way ANOVA followed by Fisher’s LSD).
